# Supplementary material for: Cyclodextrin reduces cholesterol crystal uptake by circulating monocytes in patients undergoing coronary angiography
Source: PLoS One. 2025 Dec 15;20(12):e0338635. doi: 10.1371/journal.pone.0338635 (PMC12747169; doi:10.1371/journal.pone.0338635)

S1 Fig. Experimental Setup. PBMC were stimulated with CD and incubated at 37°C for 6 hours. Afterwards they were stimulated with CC at 37°C for another 30min. CC-uptake was analyzed by Flow cytometry. (CC: Cholesterol Crystals, CD: Cyclodextrin, PBMC: Peripheral Mononuclear Cells)

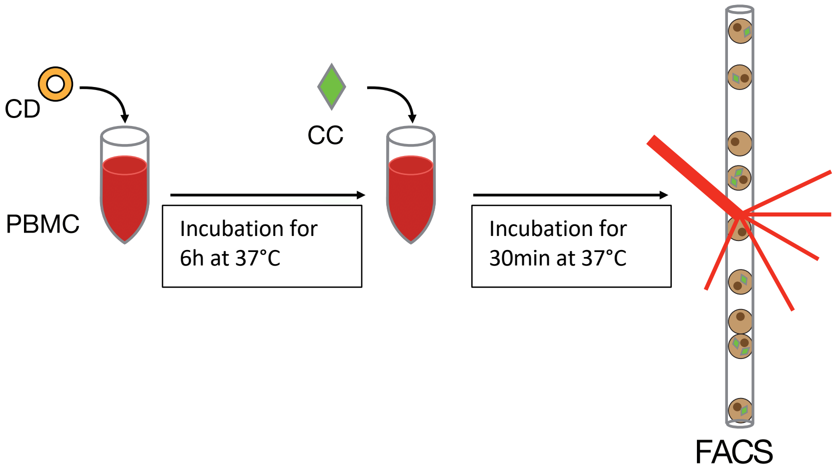

Supplement: S1 Fig — PBMC were stimulated with CD and incubated at 37°C for 6 hours. Afterwards they were stimulated with CC at 37°C for another 30 min. CC-uptake was analysed by Flow cytometry. (CC: Cholesterol Crystals, CD: Cyclodextrin, PBMC: Peripheral Mononuclear Cells). (PDF) [file pone.0338635.s001.pdf]
