# Supplementary material for: Cyclodextrin reduces cholesterol crystal uptake by circulating monocytes in patients undergoing coronary angiography
Source: PLoS One. 2025 Dec 15;20(12):e0338635. doi: 10.1371/journal.pone.0338635 (PMC12747169; doi:10.1371/journal.pone.0338635)

S2 Fig. Stimulation. 12 samples per patient – 6 stimulated with CD and 6 incubated with PBS-control. In each group triplets stimulated with CC and 3 samples incubated with PBS-control. (CD: Cyclodextrin, PBMC: Peripheral mononuclear cells, PBS: Phophat Buffered Saline)

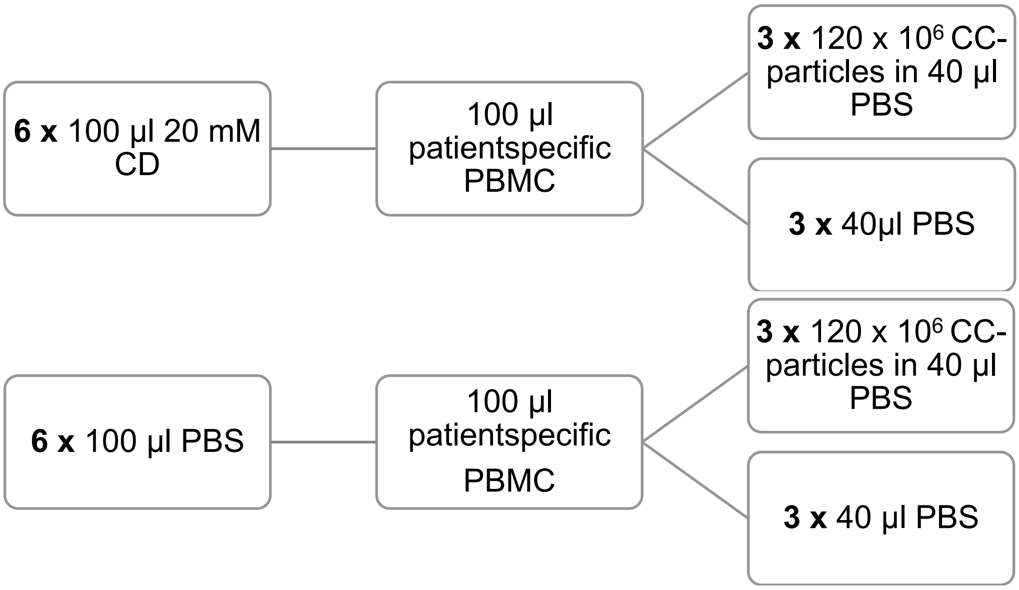

Supplement: S2 Fig — 12 samples per patient – 6 stimulated with CD and 6 incubated with PBS-control. In each group triplets stimulated with CC and 3 samples incubated with PBS-control. (CD: Cyclodextrin, PBMC: Peripheral Mononuclear Cells, PBS: Phophat Buffered Saline). (PDF) [file pone.0338635.s002.pdf]
