# Supplementary material for: Cyclodextrin reduces cholesterol crystal uptake by circulating monocytes in patients undergoing coronary angiography
Source: PLoS One. 2025 Dec 15;20(12):e0338635. doi: 10.1371/journal.pone.0338635 (PMC12747169; doi:10.1371/journal.pone.0338635)
Supplement: S1 Table — Patients with higher CC-uptake tended to have lower LDL, although this did not reach statistical significance. No differences in HDL or total cholesterol were observed in relation to CC-uptake. Data are presented as median (IQR) and groups were compared using the Mann–Whitney U test. (CC: Cholesterol Crystals, LDL: Low-Density Lipoprotein, HDL: High-Density Lipoprotein). (PDF) [file pone.0338635.s003.pdf]

S1 Table. Cholesterol levels in patients in relation to CC-uptake. Patients with higher CC-uptake tended to have lower LDL, although this did not reach statistical significance. No differences in HDL or total cholesterol were observed in relation to CC-uptake. Data are presented as median (IQR) and groups were compared using the Mann–Whitney U test. (CC: Cholesterol Crystals, LDL: Low-Density Lipoprotein, HDL: High-Density Lipoprotein)

	**Total collective**	**CC-uptake > 20 %**	**CC-uptake < 20 %**	**p-value**
	N = 76	N = 38	N = 38	
**Lab Results – Median [IQR]**				
**LDL (mg/dl)**	103.0 [80.0 – 130.8]	107.0 [80.0 – 137.7]	93.0 [79.3 – 121.0]	0.0503
**HDL (mg/dl)**	57.0 [43.5 – 64.5]	58.5 [44.8 – 65.3]	51.0 [40.0 – 64.0]	0.2528
**Total cholesterol (mg/dl)**	170.0 [136.0 – 191.5]	156.0 [129.5 – 185.5]	174.0 [145.0 – 217.0]	0.1740
