# Supplementary material for: Cyclodextrin reduces cholesterol crystal uptake by circulating monocytes in patients undergoing coronary angiography
Source: PLoS One. 2025 Dec 15;20(12):e0338635. doi: 10.1371/journal.pone.0338635 (PMC12747169; doi:10.1371/journal.pone.0338635)
Supplement: S2 Table — No differences in LDL, HDL or total cholesterol were observed across CCΔCD groups. Data are presented as median (IQR) and groups were compared using the Mann–Whitney U test. (CC: Cholesterol Crystals, LDL: Low-Density Lipoprotein, HDL: High-Density Lipoprotein). (PDF) [file pone.0338635.s004.pdf]

S2 Table. Cholesterol levels in patients in relation to CCΔCD. No differences in LDL, HDL or total cholesterol were observed across CCΔCD groups. Data are presented as median (IQR) and groups were compared using the Mann–Whitney U test. (CC: Cholesterol Crystals, LDL: Low-Density Lipoprotein, HDL: High-Density Lipoprotein)

	**Total collective**	**CCΔCD > 5.6 %**	**CCΔCD < 5.6 %**	**p-value**
	N = 76	N = 40	N = 36	
**Lab Results – Median [IQR]**				
**LDL (mg/dl)**	103.0 [80.0 – 130.8]	107.0 [80.0 – 137.7]	93.0 [79.3 – 121.0]	0.3052
**HDL (mg/dl)**	57.0 [43.5 – 64.5]	57.0 [49.0 – 66.0]	57.0 [38.3 – 64.3]	0.3812
**Total cholesterol (mg/dl)**	170.0 [136.0 – 191.5]	177.0 [130.0 – 103.0]	154.5 [138.8 – 182.5]	0.2112
