# Supplementary material for: Cyclodextrin reduces cholesterol crystal uptake by circulating monocytes in patients undergoing coronary angiography
Source: PLoS One. 2025 Dec 15;20(12):e0338635. doi: 10.1371/journal.pone.0338635 (PMC12747169; doi:10.1371/journal.pone.0338635)
Supplement: S3 Table — No significant differences in medication were observed between patients with low and high CC-uptake (all p > 0.05). Data are shown as n (%). (CC: Cholesterol Crystals, ASA: Acetylsalicylic Acid, DAPT: Dual Antiplatelet Therapy, VKA: Vitamin K Antagonist, ARB: Angiotensin receptor blocker, PPI: Proton Pump Inhibitor). (PDF) [file pone.0338635.s005.pdf]

S3 Table. Concomitant medication according to CC-uptake. No significant differences in medication were observed between patients with low and high CC-uptake (all p > 0.05). Data are shown as n (%). (CC: Cholesterol Crystals, ASA: Acetylsalicylic Acid, DAPT: Dual Antiplatelet Therapy, VKA: Vitamin K Antagonist, ARB: Angiotensin receptor blocker, PPI: Proton Pump Inhibitor)

	**Total collective**	**CC-uptake > 20 %**	**CC-uptake < 20 %**	**p-value**
	N = 76	N = 38	N = 38	
**Antiplatelet therapy – no. (%)**				
**ASA**	41 (53.9)	24 (63.2)	17 (44.7)	0.167
**P2Y12-Inhibotor**	36 (47.4)	21 (55.3)	15 (39.5)	0.251
**DAPT**	63 (82.9)	33 (86.8)	30 (78.9)	0.544
**Orale Anticoagulation – no. (%)**				
**VKA**	10 (13.2)	6 (15.8)	4 (10.5)	0.736
**DOAC**	27 (35.5)	10 (26.3)	17 (44.7)	0.150
**Cardiovascular medication – no. (%)**				
**ACE-Inhibitor/ARB**	56 (73.7)	31 (81.6)	25 (65.8)	0.192
**ß-Blocker**	65 (85.5)	34 (89.5)	31 (81.6)	0.516
**Calcium channel blocker**	13 (17.1)	8 (21.1)	5 (13.2)	0.544
**Diuretics**	54 (71.1)	27 (71.1)	27 (71.1)	1.000
**Other – no. (%)**				
**Statin**	51 (67.1)	28 (73.7)	23 (60.5)	0.329
**Oral Antidiabetics**	17 (22.4)	8 (21.1)	9 (23.7)	1.000
**Insulin**	10 (13.2)	2 (5.3)	8 (21.1)	0.086
**PPI**	50 (65.8)	25 (65.8)	25 (65.8)	1.000
**Steroids**	3 (3.9)	1 (2.6)	2 (5.3)	1.000
