# Supplementary material for: Cyclodextrin reduces cholesterol crystal uptake by circulating monocytes in patients undergoing coronary angiography
Source: PLoS One. 2025 Dec 15;20(12):e0338635. doi: 10.1371/journal.pone.0338635 (PMC12747169; doi:10.1371/journal.pone.0338635)
Supplement: S4 Table — Medication profiles were comparable across CCΔCD (all p > 0.05). Data are shown as n (%). (ASA: Acetylsalicylic Acid, DAPT: Dual Antiplatelet Therapy, VKA: Vitamin K Antagonist, ARB: Angiotensin receptor blocker, PPI: Proton Pump Inhibitor). (PDF) [file pone.0338635.s006.pdf]

**S4 Table. Concomitant medication according to CCΔCD.** Medication profiles were comparable across CCΔCD (all $p > 0.05$). Data are shown as n (%). (ASA: Acetylsalicylic Acid, DAPT: Dual Antiplatelet Therapy, VKA: Vitamin K Antagonist, ARB: Angiotensin receptor blocker, PPI: Proton Pump Inhibitor)

| | **Total collective** | **CCΔCD > 5.6 %** | **CCΔCD < 5.6 %** | **p-value** |
|---|---|---|---|---|
| | N = 76 | N = 40 | N = 36 | |
| **Antiplatelet therapy – no. (%)** | | | | |
| **ASA** | 41 (53.9) | 21 (51.2) | 20 (57.1) | 0.650 |
| **P2Y12-Inhibotor** | 36 (47.4) | 17 (41.5) | 19 (54.3) | 0.357 |
| **DAPT** | 63 (82.9) | 35 (85.4) | 28 (80.0) | 0.558 |
| **Orale Anticoagulation – no. (%)** | | | | |
| **VKA** | 10 (13.2) | 5 (12.2) | 5 (14.3) | 1.000 |
| **DOAC** | 27 (35.5) | 14 (34.1) | 13 (37.1) | 0.814 |
| **Cardiovascular medication – no. (%)** | | | | |
| **ACE-Inhibitor/ARB** | 56 (73.7) | 28 (68.3) | 28 (80.0) | 0.302 |
| **ß-Blocker** | 65 (85.5) | 35 (85.4) | 30 (85.7) | 1.000 |
| **Calcium channel blocker** | 13 (17.1) | 6 (14.6) | 7 (20.0) | 0.558 |
| **Diuretics** | 54 (71.1) | 27 (65.9) | 27 (77.1) | 0.319 |
| **Other – no. (%)** | | | | |
| **Statin** | 51 (67.1) | 26 (63.4) | 25 (71.4) | 0.476 |
| **Oral Antidiabetics** | 17 (22.4) | 9 (22.0) | 8 (22.9) | 1.000 |
| **Insulin** | 10 (13.2) | 4 (9.8) | 6 (17.1) | 0.498 |
| **PPI** | 50 (65.8) | 29 (70.7) | 21 (60.0) | 0.344 |
| **Steroids** | 3 (3.9) | 2 (4.9) | 1 (2.9) | 1.000 |
